# Supplementary material for: Factors Associated with Dog Rabies Immunization in Changsha, China: Results of a Cross-Sectional Cluster Survey, 2015–2021
Source: Viruses. 2022 Dec 31;15(1):138. doi: 10.3390/v15010138 (PMC9863250; doi:10.3390/v15010138)
Supplement: Supplementary file 1 [file viruses-15-00138-s001.zip › viruses-2014760-supplementary.pdf]

Table S1. Qualified rate of vaccine antibody in each urban area, Changsha, China, 2015-2021.

	2015	2016	2017	2018	2019	2020	2021
**Kaifu**	34.55% (19/55)	44.19% (19/43)	70.73% (87/123)	82.95% (107/129)	56.80% (71/125)	67.88% (112/165)	84.38% (81/96)
**Furong**	28.74% (25/87)	66.67% (34/51)	77.53% (138/178)	82.72% (134/162)	70.00% (63/90)	76.50% (153/200)	92.83% (246/265)
**Tianxin**	52.50% (21/40)	47.37% (18/38)	63.53% (54/85)	46.32% (44/95)	80.77% (42/52)	71.67% (86/120)	86.23% (119/138)
**Yuhua**	62.86% (66/105)	56.82% (75/132)	77.87% (190/244)	84.38% (135/160)	78.21% (122/156)	82.50% (198/240)	94.19% (292/310)
**Yuelu**	48.98% (24/49)	51.14% (45/88)	66.67% (66/99)	52.87% (92/174)	74.77% (80/107)	70.24% (118/168)	89.73% (131/146)
**Total**	46.13% (155/336)	54.26% (191/352)	73.38% (535/729)	71.11% (512/720)	71.32% (378/530)	74.69% (667/893)	90.99% (869/955)
